# (*Z*)-3′,6′-Bis(diethyl­amino)-2-(4-oxopent-2-en-2-yl­amino)­spiro­[isoindoline-1,9′-xanthen]-3-one

**DOI:** 10.1107/S1600536812027602

**Published:** 2012-06-27

**Authors:** Chang-Bin Guo, Ri-Cai He, Ai-Min Li

**Affiliations:** a Department of Chemistry, Capital Normal University, Beijing 100048, People’s Republic of China

## Abstract

In the title compound, C_33_H_38_N_4_O_3_, the mean planes of the 9*H*-xanthene unit and spiro­lactam (nine-atom) core are almost mutually perpendicular at 87.26 (6)°. Intra­molecular N—H⋯O and C—H⋯N inter­actions influence the 4-oxo­pent-2-en-2-yl­amino conformation. In the crystal, weak C—H⋯O hydrogen bonds link the mol­ecules into chains along [001].

## Related literature

For the use of Rhodamine B derivatives as fluorescent chemosensors, see: Zhang *et al.* (2007 ▶); Soh *et al.* (2007 ▶). For a related structure, see: Xiang *et al.* (2006 ▶).
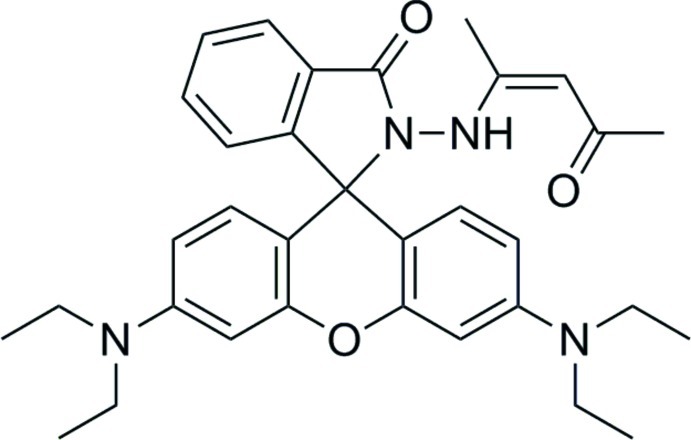


## Experimental

### 

#### Crystal data

C_33_H_38_N_4_O_3_
*M* *_r_* = 538.67
Monoclinic, 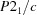

*a* = 17.4255 (3) Å
*b* = 15.0965 (3) Å
*c* = 11.5354 (2) Å
β = 103.008 (1)°
*V* = 2956.68 (9) Å^3^
*Z* = 4
Mo *K*α radiation
μ = 0.08 mm^−1^
*T* = 296 K
0.26 × 0.26 × 0.24 mm

#### Data collection

Bruker APEXII CCD area-detector diffractometer
Absorption correction: multi-scan (*SADABS*; Bruker, 2007 ▶) *T*_min_ = 0.666, *T*_max_ = 0.746
24441 measured reflections
7000 independent reflections
4223 reflections with *I* > 2σ(*I*)
*R*_int_ = 0.038

#### Refinement

*R*[*F* ^2^ > 2σ(*F* ^2^)] = 0.055
*wR*(*F* ^2^) = 0.165
*S* = 1.04
7000 reflections
365 parameters
H atoms treated by a mixture of independent and constrained refinement
Δρ_max_ = 0.31 e Å^−3^
Δρ_min_ = −0.23 e Å^−3^

### 

Data collection: *APEX2* (Bruker, 2007 ▶); cell refinement: *APEX2* and *SAINT* (Bruker, 2007 ▶); data reduction: *SAINT*; program(s) used to solve structure: *SHELXS97* (Sheldrick, 2008 ▶); program(s) used to refine structure: *SHELXL97* (Sheldrick, 2008 ▶); molecular graphics: *SHELXTL* (Sheldrick, 2008 ▶); software used to prepare material for publication: *SHELXTL* and *PLATON* (Spek, 2009 ▶).

## Supplementary Material

Crystal structure: contains datablock(s) I, global. DOI: 10.1107/S1600536812027602/gg2084sup1.cif

Structure factors: contains datablock(s) I. DOI: 10.1107/S1600536812027602/gg2084Isup2.hkl

Supplementary material file. DOI: 10.1107/S1600536812027602/gg2084Isup3.cml

Additional supplementary materials:  crystallographic information; 3D view; checkCIF report

## Figures and Tables

**Table 1:** Hydrogen-bond geometry (Å, °)

*D*—H⋯*A*	*D*—H	H⋯*A*	*D*⋯*A*	*D*—H⋯*A*
N3—H1⋯O3	0.88 (2)	1.92 (2)	2.641 (2)	139 (2)
C33—H33*A*⋯N2	0.96	2.30	2.785 (3)	110
C7—H7*B*⋯O3^i^	0.97	2.60	3.334 (3)	133

Symmetry code: (i) 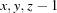.

**Fig. 1.**
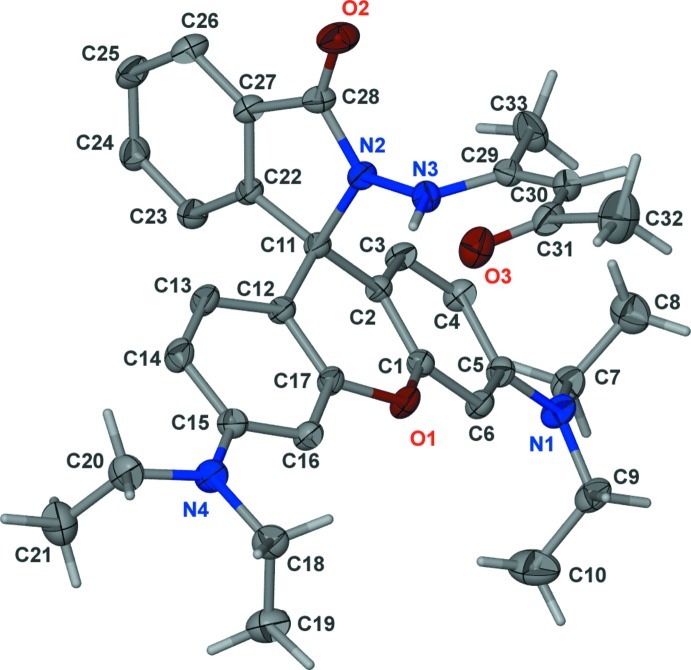
The structure of (I) with 35% probability displacement ellipsoids and the atom numbering scheme.

**Fig. 2.**
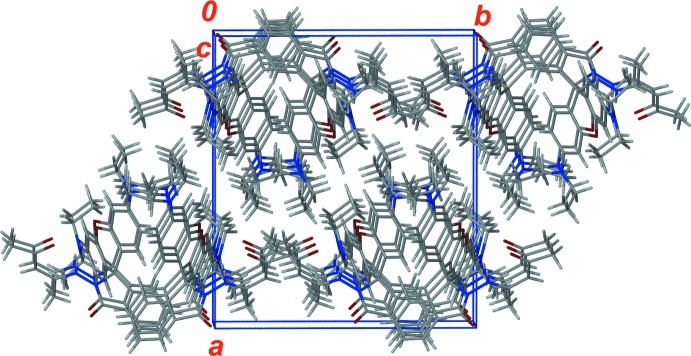
The crystal structure packing structure of (I).

## supplementary crystallographic information

### Comment

Rhodamine B derivatives are known to have excellent photophysical properties, such as the long absorption and emission wavelengths elongated to the visible region. Therefore, they have been extensively used as fluorescent chemosensors for heavy and transition metal ions, such as Cu(II) (Zhang *et al.*, 2007) and Hg(II) chemical sensor (Soh *et al.*, 2007). Herein, we report the synthesis and crystal structure of a new Rhodamine B derivative, namely (Z)-3',6'-bis(diethylamino)-2-(4-oxopent-2-en-2-ylamino)spiroisoindoline- [1,9'-xanthen]-3-one.

In the title compound C_33_H_38_N_4_O_3_, the mean plane of the 9*H*-xanthene moiety and that of the spirolactam moiety are almost perpendicular to each other (Fig. 1). Two *N*,*N*-diethylamino groups bond to the 9*H*-xanthene moiety at C5 and C15, respectivly, while a 4-oxopent-2-en-2-ylamino links to the spirolactam moiety at N2 position (Fig. 1). The 9*H*-xanthene fragment exhibits a butterfly-like conformation with two benzene rings (C12-C13-C14-C15-C16-C17 and C1-C2-C3-C4-C5-C6, respectively) exhibiting a dihedral angle of 160.06 (4)°. For the 4-oxopent-2-en-2-ylamino group, the double bond between C29 and C30 shows a Z-configuration. The structure of the title compound is similar to that of other reported Rhodamine B derivatives (Xiang *et al.* 2006). The molecular packing is stabilized by an intermolecular interaction (C7—H7B···O3^i^, C7O3^i^ = 3.334 (3) Å, C7—H7B···O3^i^ = 133 °).

### Experimental

To a solution of 2-amino-3',6'-bis(diethylamino)spiro [isoindoline-1,9'-xanthen]-3-one (0.50 g, 1.09 mmol) in 3 mL absolute anhydrous ethanol was added 0.5 mL pentane-2,4-dione and CAN (5% mol), then the resulting mixture was stirred at room temperature for 6 h. The reaction mixture was dissolved in 20 mL CH_2_Cl_2_, washed twice with water and dried over Na_2_SO_4_, filtered, and the filtrate was concentrated under reduced pressure. The residue was purified by column chromatography (EA:PE=1:1, v/v) to yield the title compound as a white powder (0.47 g, 80.1%). Crystals suitable for X-ray analysis were obtained by slow evaporation of a solution of the title compound in ethanol at room temperature in four days.

### Refinement

All the H atoms were discernible in the difference electro density maps with C—H = 0.93 and 0.96Å for aryl and methyl, respectively. U*_iso_*(H)=1.2U*_eq_*(C).

### Crystal data

**Table:**

C_33_H_38_N_4_O_3_	*F*(000) = 1152
*M**_r_* = 538.67	*D*_x_ = 1.210 Mg m^−^^3^*D*_m_ = 1.210 Mg m^−^^3^*D*_m_ measured by not measured
Monoclinic, *P*2_1_/*c*	Mo *K*α radiation, λ = 0.71073 Å
Hall symbol: -P 2ybc	Cell parameters from 7869 reflections
*a* = 17.4255 (3) Å	θ = 2.5–28.8°
*b* = 15.0965 (3) Å	µ = 0.08 mm^−^^1^
*c* = 11.5354 (2) Å	*T* = 296 K
β = 103.008 (1)°	Block, colorless
*V* = 2956.68 (9) Å^3^	0.26 × 0.26 × 0.24 mm
*Z* = 4	

### Data collection

**Table:**

Bruker APEXII CCD area-detector diffractometer	7000 independent reflections
Radiation source: fine-focus sealed tube	4223 reflections with *I* > 2σ(*I*)
Graphite monochromator	*R*_int_ = 0.038
ω scans	θ_max_ = 28.0°, θ_min_ = 1.8°
Absorption correction: multi-scan (*SADABS*; Bruker, 2007)	*h* = −22→22
*T*_min_ = 0.666, *T*_max_ = 0.746	*k* = −19→19
24441 measured reflections	*l* = −15→15

### Refinement

**Table:**

Refinement on *F*^2^	Primary atom site location: structure-invariant direct methods
Least-squares matrix: full	Secondary atom site location: difference Fourier map
*R*[*F*^2^ > 2σ(*F*^2^)] = 0.055	Hydrogen site location: inferred from neighbouring sites
*wR*(*F*^2^) = 0.165	H atoms treated by a mixture of independent and constrained refinement
*S* = 1.04	*w* = 1/[σ^2^(*F*_o_^2^) + (0.0751*P*)^2^ + 0.4182*P*] where *P* = (*F*_o_^2^ + 2*F*_c_^2^)/3
7000 reflections	(Δ/σ)_max_ < 0.001
365 parameters	Δρ_max_ = 0.31 e Å^−^^3^
0 restraints	Δρ_min_ = −0.23 e Å^−^^3^

### Special details

**Table:**

Geometry. All esds (except the esd in the dihedral angle between two l.s. planes) are estimated using the full covariance matrix. The cell esds are taken into account individually in the estimation of esds in distances, angles and torsion angles; correlations between esds in cell parameters are only used when they are defined by crystal symmetry. An approximate (isotropic) treatment of cell esds is used for estimating esds involving l.s. planes.
Refinement. Refinement of F^2^ against ALL reflections. The weighted R-factor wR and goodness of fit S are based on F^2^, conventional R-factors R are based on F, with F set to zero for negative F^2^. The threshold expression of F^2^ > 2sigma(F^2^) is used only for calculating R-factors(gt) etc. and is not relevant to the choice of reflections for refinement. R-factors based on F^2^ are statistically about twice as large as those based on F, and R- factors based on ALL data will be even larger.

### Fractional atomic coordinates and isotropic or equivalent isotropic displacement parameters (Å^2^)

**Table:**

	*x*	*y*	*z*	*U*_iso_*/*U*_eq_	
C1	0.72483 (9)	0.55285 (12)	0.35969 (17)	0.0411 (4)	
C2	0.79924 (9)	0.58683 (11)	0.40887 (16)	0.0391 (4)	
C3	0.85265 (10)	0.58468 (13)	0.33574 (18)	0.0480 (5)	
H3A	0.9032	0.6065	0.3652	0.058*	
C4	0.83408 (11)	0.55181 (14)	0.22229 (18)	0.0516 (5)	
H4A	0.8721	0.5512	0.1773	0.062*	
C5	0.75792 (10)	0.51884 (13)	0.17260 (17)	0.0464 (4)	
C6	0.70398 (10)	0.51974 (12)	0.24555 (17)	0.0461 (4)	
H6A	0.6534	0.4978	0.2168	0.055*	
C7	0.79176 (13)	0.50105 (16)	−0.0224 (2)	0.0622 (6)	
H7A	0.8178	0.5578	−0.0046	0.075*	
H7B	0.7609	0.5034	−0.1036	0.075*	
C8	0.85292 (16)	0.43034 (19)	−0.0135 (3)	0.0839 (8)	
H8A	0.8857	0.4430	−0.0681	0.126*	
H8B	0.8276	0.3741	−0.0330	0.126*	
H8C	0.8846	0.4286	0.0661	0.126*	
C9	0.65814 (12)	0.46323 (16)	0.0011 (2)	0.0661 (6)	
H9A	0.6348	0.4319	0.0580	0.079*	
H9B	0.6592	0.4230	−0.0641	0.079*	
C10	0.60719 (17)	0.5411 (2)	−0.0460 (3)	0.1075 (11)	
H10A	0.5551	0.5209	−0.0825	0.161*	
H10B	0.6291	0.5717	−0.1040	0.161*	
H10C	0.6047	0.5807	0.0182	0.161*	
C11	0.81924 (9)	0.61870 (11)	0.53530 (16)	0.0393 (4)	
C12	0.74476 (9)	0.64566 (12)	0.57294 (17)	0.0417 (4)	
C13	0.74226 (11)	0.70387 (13)	0.66550 (18)	0.0523 (5)	
H13A	0.7879	0.7342	0.7011	0.063*	
C14	0.67532 (11)	0.71844 (14)	0.70650 (19)	0.0555 (5)	
H14A	0.6761	0.7598	0.7665	0.067*	
C15	0.60520 (10)	0.67139 (13)	0.65862 (17)	0.0479 (5)	
C16	0.60620 (10)	0.61550 (12)	0.56295 (18)	0.0473 (5)	
H16A	0.5607	0.5851	0.5268	0.057*	
C17	0.67389 (10)	0.60457 (12)	0.52103 (17)	0.0423 (4)	
C18	0.46914 (11)	0.62613 (16)	0.6584 (2)	0.0612 (6)	
H18A	0.4395	0.6212	0.7199	0.073*	
H18B	0.4863	0.5670	0.6431	0.073*	
C19	0.41471 (14)	0.6596 (2)	0.5467 (3)	0.0848 (8)	
H19A	0.3709	0.6198	0.5239	0.127*	
H19B	0.4427	0.6630	0.4841	0.127*	
H19C	0.3958	0.7174	0.5610	0.127*	
C20	0.53268 (13)	0.74897 (16)	0.7899 (2)	0.0663 (6)	
H20A	0.5831	0.7544	0.8459	0.080*	
H20B	0.4942	0.7309	0.8342	0.080*	
C21	0.50958 (18)	0.83815 (19)	0.7354 (3)	0.0957 (9)	
H21A	0.5069	0.8800	0.7970	0.144*	
H21B	0.4590	0.8340	0.6814	0.144*	
H21C	0.5481	0.8575	0.6930	0.144*	
C28	0.92523 (10)	0.57242 (13)	0.69867 (18)	0.0491 (5)	
C27	0.94264 (10)	0.66190 (12)	0.65927 (17)	0.0464 (5)	
C26	1.00665 (11)	0.71638 (15)	0.7041 (2)	0.0592 (6)	
H26A	1.0454	0.6993	0.7697	0.071*	
C25	1.01124 (11)	0.79624 (15)	0.6490 (2)	0.0605 (6)	
H25A	1.0532	0.8341	0.6783	0.073*	
C24	0.95405 (11)	0.82103 (14)	0.5504 (2)	0.0569 (5)	
H24A	0.9591	0.8745	0.5127	0.068*	
C23	0.88946 (10)	0.76746 (13)	0.50691 (19)	0.0506 (5)	
H23A	0.8507	0.7848	0.4414	0.061*	
C22	0.88396 (9)	0.68792 (12)	0.56306 (17)	0.0417 (4)	
C29	0.83791 (10)	0.39074 (12)	0.59552 (18)	0.0469 (5)	
C33	0.90031 (14)	0.38742 (16)	0.5252 (3)	0.0764 (7)	
H33A	0.9179	0.4464	0.5140	0.115*	
H33B	0.8791	0.3609	0.4490	0.115*	
H33C	0.9440	0.3528	0.5673	0.115*	
C30	0.80224 (11)	0.31622 (13)	0.6247 (2)	0.0559 (5)	
H30A	0.8153	0.2625	0.5946	0.067*	
C31	0.74681 (12)	0.31462 (15)	0.6976 (2)	0.0601 (6)	
C32	0.72173 (19)	0.22546 (19)	0.7353 (3)	0.1099 (11)	
H32A	0.6843	0.2336	0.7840	0.165*	
H32B	0.7669	0.1944	0.7798	0.165*	
H32C	0.6980	0.1916	0.6660	0.165*	
N1	0.73857 (9)	0.48717 (13)	0.05820 (15)	0.0580 (5)	
N2	0.85909 (8)	0.54639 (10)	0.61756 (14)	0.0445 (4)	
N3	0.81807 (9)	0.47045 (10)	0.63140 (15)	0.0463 (4)	
N4	0.53859 (9)	0.67999 (12)	0.70403 (16)	0.0569 (5)	
O1	0.66609 (7)	0.55024 (9)	0.42302 (12)	0.0517 (4)	
O2	0.96133 (9)	0.52827 (11)	0.78133 (15)	0.0744 (5)	
O3	0.71972 (9)	0.38277 (11)	0.73323 (15)	0.0710 (5)	
H1	0.7807 (13)	0.4688 (15)	0.671 (2)	0.070 (7)*	

### Atomic displacement parameters (Å^2^)

**Table:**

	*U*^11^	*U*^22^	*U*^33^	*U*^12^	*U*^13^	*U*^23^
C1	0.0388 (8)	0.0427 (9)	0.0429 (11)	−0.0010 (7)	0.0111 (8)	−0.0032 (9)
C2	0.0391 (8)	0.0402 (9)	0.0368 (10)	−0.0009 (7)	0.0060 (7)	−0.0023 (8)
C3	0.0387 (8)	0.0578 (11)	0.0466 (12)	−0.0077 (8)	0.0078 (8)	−0.0049 (10)
C4	0.0479 (10)	0.0644 (12)	0.0455 (12)	−0.0067 (9)	0.0172 (9)	−0.0065 (10)
C5	0.0504 (10)	0.0489 (10)	0.0399 (11)	−0.0005 (8)	0.0100 (9)	−0.0055 (9)
C6	0.0417 (9)	0.0512 (11)	0.0442 (12)	−0.0061 (8)	0.0072 (8)	−0.0094 (9)
C7	0.0704 (13)	0.0709 (14)	0.0463 (13)	−0.0035 (11)	0.0156 (10)	−0.0087 (11)
C8	0.0891 (17)	0.0923 (19)	0.0758 (19)	0.0133 (14)	0.0305 (15)	−0.0065 (15)
C9	0.0631 (12)	0.0859 (17)	0.0471 (13)	−0.0102 (11)	0.0073 (11)	−0.0177 (12)
C10	0.0870 (18)	0.146 (3)	0.081 (2)	0.0269 (19)	0.0007 (16)	0.011 (2)
C11	0.0376 (8)	0.0405 (9)	0.0380 (10)	−0.0016 (7)	0.0047 (7)	−0.0005 (8)
C12	0.0394 (8)	0.0442 (10)	0.0407 (11)	−0.0020 (7)	0.0073 (8)	−0.0033 (9)
C13	0.0481 (10)	0.0574 (12)	0.0509 (13)	−0.0086 (8)	0.0101 (9)	−0.0171 (10)
C14	0.0564 (11)	0.0600 (12)	0.0521 (13)	−0.0046 (9)	0.0167 (10)	−0.0203 (11)
C15	0.0461 (9)	0.0549 (11)	0.0430 (12)	0.0032 (8)	0.0108 (8)	−0.0039 (10)
C16	0.0407 (8)	0.0547 (11)	0.0461 (12)	−0.0053 (8)	0.0090 (8)	−0.0097 (10)
C17	0.0444 (9)	0.0441 (10)	0.0384 (11)	−0.0029 (7)	0.0092 (8)	−0.0081 (9)
C18	0.0515 (10)	0.0751 (15)	0.0618 (15)	−0.0025 (10)	0.0229 (11)	−0.0047 (12)
C19	0.0626 (13)	0.108 (2)	0.0784 (19)	−0.0008 (13)	0.0049 (13)	−0.0022 (17)
C20	0.0628 (12)	0.0779 (15)	0.0641 (15)	0.0054 (11)	0.0270 (11)	−0.0147 (13)
C21	0.106 (2)	0.0736 (18)	0.105 (2)	0.0087 (15)	0.0189 (18)	−0.0119 (17)
C28	0.0422 (9)	0.0573 (12)	0.0446 (12)	−0.0013 (8)	0.0029 (9)	0.0054 (10)
C27	0.0408 (8)	0.0536 (11)	0.0434 (11)	−0.0055 (8)	0.0066 (8)	−0.0021 (9)
C26	0.0455 (10)	0.0753 (15)	0.0516 (13)	−0.0125 (9)	−0.0001 (9)	−0.0002 (12)
C25	0.0480 (10)	0.0632 (13)	0.0693 (16)	−0.0180 (9)	0.0113 (11)	−0.0093 (12)
C24	0.0572 (11)	0.0474 (11)	0.0680 (15)	−0.0082 (9)	0.0182 (11)	−0.0020 (11)
C23	0.0487 (10)	0.0474 (11)	0.0532 (13)	−0.0014 (8)	0.0062 (9)	0.0025 (10)
C22	0.0384 (8)	0.0458 (10)	0.0397 (11)	−0.0030 (7)	0.0064 (8)	−0.0049 (9)
C29	0.0438 (9)	0.0476 (11)	0.0469 (12)	0.0064 (8)	0.0049 (8)	−0.0012 (9)
C33	0.0751 (14)	0.0654 (14)	0.099 (2)	0.0142 (11)	0.0411 (14)	−0.0054 (14)
C30	0.0557 (11)	0.0431 (11)	0.0652 (15)	0.0015 (8)	0.0058 (10)	−0.0049 (10)
C31	0.0600 (11)	0.0571 (13)	0.0596 (15)	−0.0134 (10)	0.0058 (11)	−0.0025 (12)
C32	0.126 (2)	0.0762 (18)	0.134 (3)	−0.0367 (17)	0.043 (2)	0.007 (2)
N1	0.0552 (9)	0.0793 (12)	0.0401 (10)	−0.0082 (8)	0.0122 (8)	−0.0151 (9)
N2	0.0445 (7)	0.0429 (8)	0.0433 (9)	−0.0051 (6)	0.0039 (7)	0.0028 (7)
N3	0.0479 (8)	0.0430 (9)	0.0508 (10)	−0.0037 (7)	0.0166 (8)	−0.0003 (8)
N4	0.0488 (8)	0.0699 (11)	0.0556 (11)	0.0002 (8)	0.0190 (8)	−0.0136 (9)
O1	0.0432 (6)	0.0642 (8)	0.0505 (8)	−0.0143 (6)	0.0164 (6)	−0.0221 (7)
O2	0.0680 (9)	0.0804 (11)	0.0629 (11)	−0.0071 (8)	−0.0106 (8)	0.0246 (9)
O3	0.0750 (9)	0.0730 (11)	0.0710 (12)	−0.0168 (8)	0.0293 (9)	−0.0126 (9)

### Geometric parameters (Å, º)

**Table:**

C1—C6	1.378 (3)	C18—C19	1.505 (3)
C1—O1	1.386 (2)	C18—H18A	0.9700
C1—C2	1.391 (2)	C18—H18B	0.9700
C2—C3	1.390 (2)	C19—H19A	0.9600
C2—C11	1.500 (3)	C19—H19B	0.9600
C3—C4	1.369 (3)	C19—H19C	0.9600
C3—H3A	0.9300	C20—N4	1.456 (3)
C4—C5	1.412 (3)	C20—C21	1.502 (4)
C4—H4A	0.9300	C20—H20A	0.9700
C5—N1	1.372 (2)	C20—H20B	0.9700
C5—C6	1.396 (2)	C21—H21A	0.9600
C6—H6A	0.9300	C21—H21B	0.9600
C7—N1	1.468 (3)	C21—H21C	0.9600
C7—C8	1.496 (3)	C28—O2	1.216 (2)
C7—H7A	0.9700	C28—N2	1.368 (2)
C7—H7B	0.9700	C28—C27	1.478 (3)
C8—H8A	0.9600	C27—C22	1.386 (3)
C8—H8B	0.9600	C27—C26	1.389 (3)
C8—H8C	0.9600	C26—C25	1.374 (3)
C9—N1	1.454 (3)	C26—H26A	0.9300
C9—C10	1.500 (4)	C25—C24	1.385 (3)
C9—H9A	0.9700	C25—H25A	0.9300
C9—H9B	0.9700	C24—C23	1.385 (3)
C10—H10A	0.9600	C24—H24A	0.9300
C10—H10B	0.9600	C23—C22	1.378 (3)
C10—H10C	0.9600	C23—H23A	0.9300
C11—N2	1.509 (2)	C29—N3	1.343 (2)
C11—C12	1.514 (2)	C29—C30	1.364 (3)
C11—C22	1.518 (2)	C29—C33	1.497 (3)
C12—C13	1.391 (3)	C33—H33A	0.9600
C12—C17	1.391 (2)	C33—H33B	0.9600
C13—C14	1.371 (3)	C33—H33C	0.9600
C13—H13A	0.9300	C30—C31	1.416 (3)
C14—C15	1.414 (3)	C30—H30A	0.9300
C14—H14A	0.9300	C31—O3	1.240 (3)
C15—N4	1.383 (2)	C31—C32	1.509 (3)
C15—C16	1.392 (3)	C32—H32A	0.9600
C16—C17	1.382 (2)	C32—H32B	0.9600
C16—H16A	0.9300	C32—H32C	0.9600
C17—O1	1.379 (2)	N2—N3	1.379 (2)
C18—N4	1.455 (3)	N3—H1	0.88 (2)
			
C6—C1—O1	115.22 (14)	C18—C19—H19B	109.5
C6—C1—C2	122.99 (16)	H19A—C19—H19B	109.5
O1—C1—C2	121.79 (16)	C18—C19—H19C	109.5
C3—C2—C1	115.71 (16)	H19A—C19—H19C	109.5
C3—C2—C11	123.82 (14)	H19B—C19—H19C	109.5
C1—C2—C11	120.39 (15)	N4—C20—C21	114.2 (2)
C4—C3—C2	122.84 (16)	N4—C20—H20A	108.7
C4—C3—H3A	118.6	C21—C20—H20A	108.7
C2—C3—H3A	118.6	N4—C20—H20B	108.7
C3—C4—C5	120.94 (17)	C21—C20—H20B	108.7
C3—C4—H4A	119.5	H20A—C20—H20B	107.6
C5—C4—H4A	119.5	C20—C21—H21A	109.5
N1—C5—C6	122.01 (16)	C20—C21—H21B	109.5
N1—C5—C4	121.20 (16)	H21A—C21—H21B	109.5
C6—C5—C4	116.79 (17)	C20—C21—H21C	109.5
C1—C6—C5	120.72 (16)	H21A—C21—H21C	109.5
C1—C6—H6A	119.6	H21B—C21—H21C	109.5
C5—C6—H6A	119.6	O2—C28—N2	125.88 (18)
N1—C7—C8	113.3 (2)	O2—C28—C27	129.50 (18)
N1—C7—H7A	108.9	N2—C28—C27	104.58 (16)
C8—C7—H7A	108.9	C22—C27—C26	121.10 (18)
N1—C7—H7B	108.9	C22—C27—C28	109.86 (15)
C8—C7—H7B	108.9	C26—C27—C28	129.03 (18)
H7A—C7—H7B	107.7	C25—C26—C27	118.22 (19)
C7—C8—H8A	109.5	C25—C26—H26A	120.9
C7—C8—H8B	109.5	C27—C26—H26A	120.9
H8A—C8—H8B	109.5	C26—C25—C24	120.76 (18)
C7—C8—H8C	109.5	C26—C25—H25A	119.6
H8A—C8—H8C	109.5	C24—C25—H25A	119.6
H8B—C8—H8C	109.5	C23—C24—C25	121.02 (19)
N1—C9—C10	113.6 (2)	C23—C24—H24A	119.5
N1—C9—H9A	108.8	C25—C24—H24A	119.5
C10—C9—H9A	108.8	C22—C23—C24	118.40 (18)
N1—C9—H9B	108.8	C22—C23—H23A	120.8
C10—C9—H9B	108.8	C24—C23—H23A	120.8
H9A—C9—H9B	107.7	C23—C22—C27	120.44 (16)
C9—C10—H10A	109.5	C23—C22—C11	128.89 (16)
C9—C10—H10B	109.5	C27—C22—C11	110.67 (15)
H10A—C10—H10B	109.5	N3—C29—C30	119.99 (17)
C9—C10—H10C	109.5	N3—C29—C33	117.77 (17)
H10A—C10—H10C	109.5	C30—C29—C33	122.24 (18)
H10B—C10—H10C	109.5	C29—C33—H33A	109.5
C2—C11—N2	110.65 (14)	C29—C33—H33B	109.5
C2—C11—C12	109.78 (13)	H33A—C33—H33B	109.5
N2—C11—C12	108.48 (14)	C29—C33—H33C	109.5
C2—C11—C22	115.13 (15)	H33A—C33—H33C	109.5
N2—C11—C22	98.65 (13)	H33B—C33—H33C	109.5
C12—C11—C22	113.49 (14)	C29—C30—C31	124.77 (19)
C13—C12—C17	115.72 (16)	C29—C30—H30A	117.6
C13—C12—C11	124.54 (15)	C31—C30—H30A	117.6
C17—C12—C11	119.46 (16)	O3—C31—C30	122.95 (19)
C14—C13—C12	122.82 (17)	O3—C31—C32	119.2 (2)
C14—C13—H13A	118.6	C30—C31—C32	117.8 (2)
C12—C13—H13A	118.6	C31—C32—H32A	109.5
C13—C14—C15	120.80 (18)	C31—C32—H32B	109.5
C13—C14—H14A	119.6	H32A—C32—H32B	109.5
C15—C14—H14A	119.6	C31—C32—H32C	109.5
N4—C15—C16	121.23 (16)	H32A—C32—H32C	109.5
N4—C15—C14	122.02 (17)	H32B—C32—H32C	109.5
C16—C15—C14	116.75 (16)	C5—N1—C9	121.71 (16)
C17—C16—C15	120.92 (16)	C5—N1—C7	120.39 (16)
C17—C16—H16A	119.5	C9—N1—C7	115.47 (17)
C15—C16—H16A	119.5	C28—N2—N3	122.57 (16)
O1—C17—C16	115.08 (15)	C28—N2—C11	114.82 (14)
O1—C17—C12	122.17 (15)	N3—N2—C11	119.79 (13)
C16—C17—C12	122.76 (17)	C29—N3—N2	122.22 (16)
N4—C18—C19	115.8 (2)	C29—N3—H1	114.1 (15)
N4—C18—H18A	108.3	N2—N3—H1	123.3 (15)
C19—C18—H18A	108.3	C15—N4—C18	120.76 (16)
N4—C18—H18B	108.3	C15—N4—C20	121.22 (17)
C19—C18—H18B	108.3	C18—N4—C20	117.73 (16)
H18A—C18—H18B	107.4	C17—O1—C1	117.87 (13)
C18—C19—H19A	109.5		
			
C6—C1—C2—C3	−0.7 (3)	C26—C27—C22—C23	2.4 (3)
O1—C1—C2—C3	179.48 (16)	C28—C27—C22—C23	−176.69 (17)
C6—C1—C2—C11	−177.65 (17)	C26—C27—C22—C11	−177.41 (17)
O1—C1—C2—C11	2.5 (3)	C28—C27—C22—C11	3.5 (2)
C1—C2—C3—C4	0.3 (3)	C2—C11—C22—C23	53.6 (2)
C11—C2—C3—C4	177.12 (18)	N2—C11—C22—C23	171.29 (18)
C2—C3—C4—C5	0.8 (3)	C12—C11—C22—C23	−74.1 (2)
C3—C4—C5—N1	178.90 (19)	C2—C11—C22—C27	−126.66 (17)
C3—C4—C5—C6	−1.4 (3)	N2—C11—C22—C27	−8.93 (18)
O1—C1—C6—C5	179.87 (17)	C12—C11—C22—C27	105.64 (18)
C2—C1—C6—C5	0.0 (3)	N3—C29—C30—C31	−3.6 (3)
N1—C5—C6—C1	−179.30 (19)	C33—C29—C30—C31	175.6 (2)
C4—C5—C6—C1	1.0 (3)	C29—C30—C31—O3	7.0 (4)
C3—C2—C11—N2	−82.0 (2)	C29—C30—C31—C32	−171.5 (2)
C1—C2—C11—N2	94.69 (18)	C6—C5—N1—C9	8.0 (3)
C3—C2—C11—C12	158.27 (17)	C4—C5—N1—C9	−172.3 (2)
C1—C2—C11—C12	−25.0 (2)	C6—C5—N1—C7	169.48 (19)
C3—C2—C11—C22	28.7 (2)	C4—C5—N1—C7	−10.9 (3)
C1—C2—C11—C22	−154.56 (16)	C10—C9—N1—C5	79.8 (3)
C2—C11—C12—C13	−157.51 (18)	C10—C9—N1—C7	−82.5 (3)
N2—C11—C12—C13	81.5 (2)	C8—C7—N1—C5	87.5 (3)
C22—C11—C12—C13	−27.1 (3)	C8—C7—N1—C9	−109.9 (2)
C2—C11—C12—C17	28.9 (2)	O2—C28—N2—N3	10.4 (3)
N2—C11—C12—C17	−92.1 (2)	C27—C28—N2—N3	−171.76 (15)
C22—C11—C12—C17	159.33 (17)	O2—C28—N2—C11	171.30 (19)
C17—C12—C13—C14	2.0 (3)	C27—C28—N2—C11	−10.9 (2)
C11—C12—C13—C14	−171.81 (19)	C2—C11—N2—C28	133.47 (16)
C12—C13—C14—C15	2.5 (3)	C12—C11—N2—C28	−106.05 (17)
C13—C14—C15—N4	175.21 (19)	C22—C11—N2—C28	12.38 (19)
C13—C14—C15—C16	−4.7 (3)	C2—C11—N2—N3	−65.08 (19)
N4—C15—C16—C17	−177.43 (18)	C12—C11—N2—N3	55.4 (2)
C14—C15—C16—C17	2.5 (3)	C22—C11—N2—N3	173.83 (15)
C15—C16—C17—O1	−177.51 (18)	C30—C29—N3—N2	171.54 (17)
C15—C16—C17—C12	2.0 (3)	C33—C29—N3—N2	−7.6 (3)
C13—C12—C17—O1	175.24 (18)	C28—N2—N3—C29	−85.6 (2)
C11—C12—C17—O1	−10.6 (3)	C11—N2—N3—C29	114.41 (19)
C13—C12—C17—C16	−4.3 (3)	C16—C15—N4—C18	4.3 (3)
C11—C12—C17—C16	169.85 (17)	C14—C15—N4—C18	−175.7 (2)
O2—C28—C27—C22	−178.0 (2)	C16—C15—N4—C20	−169.4 (2)
N2—C28—C27—C22	4.3 (2)	C14—C15—N4—C20	10.7 (3)
O2—C28—C27—C26	3.0 (4)	C19—C18—N4—C15	−83.0 (3)
N2—C28—C27—C26	−174.7 (2)	C19—C18—N4—C20	90.9 (2)
C22—C27—C26—C25	−1.3 (3)	C21—C20—N4—C15	81.3 (3)
C28—C27—C26—C25	177.6 (2)	C21—C20—N4—C18	−92.6 (3)
C27—C26—C25—C24	−0.9 (3)	C16—C17—O1—C1	164.94 (16)
C26—C25—C24—C23	2.1 (3)	C12—C17—O1—C1	−14.6 (3)
C25—C24—C23—C22	−1.1 (3)	C6—C1—O1—C17	−160.98 (17)
C24—C23—C22—C27	−1.2 (3)	C2—C1—O1—C17	18.9 (2)
C24—C23—C22—C11	178.60 (18)		

### Hydrogen-bond geometry (Å, º)

**Table:**

*D*—H···*A*	*D*—H	H···*A*	*D*···*A*	*D*—H···*A*
N3—H1···O3	0.88 (2)	1.92 (2)	2.641 (2)	139 (2)
C33—H33*A*···N2	0.96	2.30	2.785 (3)	110
C7—H7*B*···O3^i^	0.97	2.60	3.334 (3)	133

Symmetry code: (i) *x*, *y*, *z*−1.
